# Correction: Top ten research priorities for Essential Emergency and Critical Care: A modified Delphi process

**DOI:** 10.1371/journal.pgph.0007403

**Published:** 2026-09-29

**Authors:** Ryan Rhys Ellis, Wurry Ayuningtyas, Alexandra Wharton Smith, Martin Gerdin Wärnberg, Carl Otto Schell, The EECC Prioritisation Group, Tim Baker

S1 File was uploaded incorrectly. Please see the correct S1 File here.

## Supporting information

S1 File Expert participants. (DOCX)

## References

[1] Ellis RR, Ayuningtyas W, Wharton Smith A, Gerdin Wärnberg M, Otto Schell C, The EECC Prioritisation Group, et al. Top ten research priorities for Essential Emergency and Critical Care: A modified Delphi process. PLOS Glob Public Health. 2026;6(3):e0005262. doi: 10.1371/journal.pgph.0005262 41801881 PMC12970883

