# Supplementary material for: Top ten research priorities for Essential Emergency and Critical Care: A modified Delphi process
Source: PLOS Glob Public Health. 2026 Mar 9;6(3):e0005262. doi: 10.1371/journal.pgph.0005262 (PMC12970883; doi:10.1371/journal.pgph.0005262)
Supplement: S1 File — (DOCX) [file pgph.0005262.s001.docx]

**S1: Expert Participants and Backgrounds**

This supplement lists the experts who participated in the Essential Emergency and Critical Care (EECC) global research priority-setting exercise, along with brief biographical summaries. Biographical details were extracted from the conference participant booklet provided to attendees.

**Organising Committee**

Anna Hvarfner – Medical doctor and PhD student in Global Public Health at Karolinska Institutet; engaged in EECC research with experience from Tanzania and Sweden.

Tim Baker – Associate Professor and Critical Care Physician/Anesthesiologist; over 20 years’ experience in global critical care research and implementation.

Christoffer Hintze – IT professional supporting digital systems and logistics during the EECC conference.

**Expert Participants**

Global Health & Critical Care Researchers

Alexandra Wharton-Smith – Global health researcher and technical adviser; PhD candidate at LSHTM focusing on EECC.

Andreas Barratt-Due – Senior consultant in anesthesiology and critical care at Oslo University Hospital.

Adeoye – Professor and Chair of Emergency Medicine, Washington University St. Louis; trauma and emergency medicine expert.

Andreas Wellhagen – Anesthesiologist, Nyköpings Lasarett, Sweden; involved in EECC teaching and research.

Carina King – Epidemiologist at Karolinska Institutet; specialist in implementation research and medical oxygen systems.

Jamie Rylance – Physician in pulmonary/internal medicine; WHO adviser on emergency clinical management and epidemic response.

Jonas Blixt – Neuro ICU clinician at Karolinska University Hospital; researcher on trauma and ischemia.

Kjell Arne Johansson – Professor of medical ethics and health economics, University of Bergen.

Claudia Hanson – Professor in Global Public Health at LSHTM/Karolinska Institutet.

Margaret E. Kruk – Professor of Health Systems; global expert in health system performance.

Nobhojit Roy – Professor and Chair of Global Surgery & Trauma; former WHO Technical Officer.

Petronella Bjurling-Sjöberg – Senior lecturer and ICU specialist nurse, Sweden.

Terese Beyene – Associate Professor and Emergency Medicine specialist, Addis Ababa University.

Clinicians & Critical Care Practitioners

Alhassan Datti Mohammed – Consultant anaesthetist and intensivist in Nigeria.

Emily Tegnell – Specialist registrar in anesthesiology and ICU medicine, Sweden.

Godfrey Barabona – Clinician-researcher in Tanzania; EECC trainer and advocate.

Hauro Ndulu – Consultant anesthesiologist at Muhimbili National Hospital.

Louise Lorentzen – Consultant anesthesiologist in Nyköping, Sweden.

Ryan Ellis – Anesthesiology resident at Örebro University Hospital.

Celia Blaas Kia Ora – Anaesthesiologist from New Zealand with EECC implementation experience.

Awny Auniyatus – Paediatrician from Surabaya, Indonesia.

Nora Zergi – Anesthesiology and ICU specialist, Haukeland University Hospital.

Health Systems, Policy & Implementation Experts

Kumanan Rasanathan – WHO/Alliance for Health Policy and Systems Research; expert in equity and implementation.

Ulrika Baker – Medical doctor and public health specialist with experience across Africa and Asia.

Alexa Wharton-Smith – EECC Network communications and stakeholder engagement lead.

Martin Gerdin – Senior researcher in global health and priority-setting at Karolinska Institutet.

Junior Researchers & Trainees

Simon-Fredrick Schell – Medical student with EECC research experience.

Søren Schoder – Anesthesiologist in Denmark with global anesthesia involvement.

Nick Leen – Medical professional with experience in digital communication and global health.
