# Supplementary material for: Top ten research priorities for Essential Emergency and Critical Care: A modified Delphi process
Source: PLOS Glob Public Health. 2026 Mar 9;6(3):e0005262. doi: 10.1371/journal.pgph.0005262 (PMC12970883; doi:10.1371/journal.pgph.0005262)
Supplement: S1 Data — (DOCX) [file pgph.0005262.s002.docx]

S2: Quantitative data with ranked EECC research priorities by mean Likert Score

| Rank | Question No. | EECC Research Priority Question | n | Mean | SD | Median | IQR |
| --- | --- | --- | --- | --- | --- | --- | --- |
| 1 | 8 | What are the underlying determinants of whether EECC is provided or not? | 41 | 3.41 | 1.05 | 4 | 1 |
| 2 | 15 | What are the impacts of EECC on short and long-term mortality and morbidity? | 41 | 3.39 | 1.04 | 4 | 1 |
| 3 | 1 | What is the global burden of critical illness? | 41 | 3.34 | 1.11 | 4 | 1 |
| 4 | 11 | What are the facilitators and barriers for healthcare workers to implement and sustain EECC? | 41 | 3.32 | 1.07 | 4 | 1 |
| 5 | 18 | What is the impact of implementing EECC on reducing preventable in-hospital mortality among critically ill patients? | 41 | 3.27 | 1.06 | 4 | 1 |
| 6 | 25 | What are the key indicators of EECC adherence? | 41 | 3.22 | 1.02 | 3 | 1 |
| 7 | 4 | What are the current provisions for critical care, the existing gaps, prevailing clinical practices, knowledge levels among healthcare providers, and the overall readiness of health facilities to deliver essential critical care services? | 41 | 3.05 | 1.01 | 3 | 1 |
| 8 | 23 | What are the core outcomes we should measure in EECC research? | 41 | 3.05 | 1 | 3 | 1 |
| 9 | 16 | What is the impact of EECC on outcomes in different patient subgroups? | 41 | 3.02 | 1.19 | 3 | 2 |
| 10 | 17 | What is the cost, cost-effectiveness, and budget impact of EECC? | 41 | 3.02 | 1.18 | 3 | 2 |
| 11 | 10 | Should a reduced EECC package be developed for resource-deficient settings, e.g. pre-hospital and community settings? | 41 | 2.93 | 1.18 | 3 | 2 |
| 12 | 7 | How might conflict, climate catastrophes, pandemics and public health emergencies impact the critical illness burden and the burden on the health system? | 41 | 2.9 | 1.22 | 3 | 2 |
| 13 | 13 | Which of the 40 defined EECC treatments and actions have the greatest impact on mortality and morbidity, and which are most cost-effective? | 41 | 2.85 | 1.22 | 3 | 2 |
| 14 | 21 | What are the broader impacts of implementing EECC, including unintentional consequences, for patients, providers, health systems, communities and the environment? | 41 | 2.83 | 1.21 | 3 | 2 |
| 15 | 12 | How can the components of EECC be operationalised in current policies, guidelines and curricula in different contexts? | 41 | 2.8 | 1.2 | 3 | 2 |
| 16 | 27 | Can AI be used to extract medical records of treatments provided to critically ill patients to inform modifications to EECC? | 41 | 2.76 | 1.17 | 3 | 2 |
| 17 | 28 | How best can foundational EECC be linked to more advanced critical care and definitive care programmes? | 41 | 2.76 | 1.16 | 3 | 2 |
| 18 | 3 | What are the perceptions about critical illness, critical care, and EECC among different groups (patients, caregivers, health workers, hospital management, health authorities, policy-makers, global stakeholders)? | 41 | 2.73 | 1.02 | 3 | 1 |
| 19 | 2 | What are the best definitions and criteria to measure critical illness? | 41 | 2.68 | 1.23 | 3 | 2 |
| 20 | 22 | Should the wording in Essential Emergency and Critical Care be changed to promote greater universal understanding of the concept? | 41 | 2.63 | 1.19 | 3 | 2 |
| 21 | 24 | Should EECC be differentiated for different population age groups, i.e., neonatal, paediatric and adult? | 41 | 2.61 | 1.01 | 3 | 1 |
| 22 | 9 | What is the perceived value (cost, appropriateness, acceptability, feasibility) of EECC for stakeholders across different levels? | 41 | 2.59 | 1.19 | 3 | 2 |
| 23 | 19 | What is the sustainability of EECC implementation? | 41 | 2.46 | 0.99 | 3 | 1 |
| 24 | 5 | What are the ethical considerations, norms and practices around care for critically ill patients? | 41 | 2.44 | 0.95 | 2 | 1 |
| 25 | 14 | What can we learn from implementing other EECC-related initiatives, implementation models and quality improvement science? | 41 | 2.34 | 1.15 | 3 | 2 |
| 26 | 26 | Is a revision of the EECC package necessary at this stage, or should it be scheduled for a future date, and if so, when should this be? | 41 | 2.29 | 1.13 | 2 | 2 |
| 27 | 20 | How does EECC implementation affect patient flows through the health systems? | 41 | 2.1 | 1.09 | 2 | 2 |
| 28 | 6 | In selected settings, what is the political economy of EECC (governance, finance, interest groups)? | 41 | 2.07 | 1.1 | 2 | 2 |
