# Supplementary material for: Top ten research priorities for Essential Emergency and Critical Care: A modified Delphi process
Source: PLOS Glob Public Health. 2026 Mar 9;6(3):e0005262. doi: 10.1371/journal.pgph.0005262 (PMC12970883; doi:10.1371/journal.pgph.0005262)
Supplement: Attachment — Submitted filename: PLOS EECC Review 2 (1).pdf [file pgph.0005262.s004.pdf]

This manuscript has undergone substantial revision, and I commend the authors for their good-faith efforts to address the majority of the concerns raised in the initial review. The study remains a methodologically sound and important contribution to the development of the EECC research agenda. Consequently, my overall recommendation is Minor Revision Required, contingent upon the authors addressing the remaining critical issues.

The revised manuscript demonstrates improved clarity and has successfully addressed several key methodological and reporting requirements from Round 1 as described below:

- The Introduction now clearly explains the context and rationale for the EECC priority-setting process, explicitly stating the gap left by previous agendas focused on intensive care.
- The EECC definition is much clearer for non-specialists, with explicit inclusion and exclusion criteria.
- Methodological details regarding the **Group Assignment Process** (purposive assignment for balance of expertise, gender, and regional background) and the **Rationale for Software Choice** have been adequately incorporated.
- Ethical justification has been sufficiently addressed by citing appropriate precedents for similar consensus-based exercises.
- Formatting issues, such as the addition of percentages to Table 1, have been fully addressed.

Critical Revisions: Data Transparency

Despite these improvements, the omission of the quantitative scoring data that underpins the final priority rankings is lacking. The authors' current stance—that their focus was solely on identifying a top ten set rather than quantifying consensus—is an insufficient justification for withholding this data. Without the summary statistics for all 28 questions (Mean Score, Standard Deviation, Median, and Interquartile Range):Readers cannot assess the strength of consensus or the robustness of the rankings; It is impossible to determine whether the final Top 10 list represents a natural grouping or an arbitrary cutoff point; The findings cannot be independently validated. ***This lack of transparency violates PLOS data availability standards and prevents a full assessment of whether the conclusions are supported by the data.***

- **Mandatory Requirement for Acceptance**: The authors must create a supplementary table providing the quantitative scoring data for all 28 questions. Furthermore, the Data Availability Statement on pages 2-3 must be revised to accurately reflect that survey responses (Likert scores) exist, even if only aggregated statistics are being shared.

Several other points were only partially addressed or require greater more reflection:

1. Geographic and Stakeholder Representation**:** The authors must explicitly state the 9-country limitation (primarily high-income settings) in the abstract, as suggested in the first round. In the discussion, the authors should explain why key stakeholders, such as

patient and community representatives, were excluded from the initial priority-setting process.

2. Refinement Theme Absence: The discussion on why no top-ranked questions emerged from the 'refining' theme remains superficial. The authors need to critically acknowledge the potential for selection bias, as inviting experts already invested in the existing EECC framework may have influenced the lack of refinement priorities.
3. Methods Description: The description of the selection process, where "Each group selected the seven most important research questions," is incomplete. The authors must clarify the mechanism used for this selection-e.g. was it consensus, voting, or individual ranking within the groups?
4. Critical Reflection on Methods: The discussion on the methods potential impact on results remains minimal. The authors should offer a more critical reflection on:
    a. Whether dominance patterns were observed during initial in-person group discussions.
    b. How the compressed two-day format may have limited deep deliberation on complex, cross-cutting issues.
    c. The potential silo effects created by thematic grouping.
5. Equity, Feasibility, and Urgency: The manuscript mentions these important criteria in the conclusion but must explicitly acknowledge as a limitation that these factors were not systematically integrated into the scoring or prioritization framework of this study.
