# Supplementary material for: Top ten research priorities for Essential Emergency and Critical Care: A modified Delphi process
Source: PLOS Glob Public Health. 2026 Mar 9;6(3):e0005262. doi: 10.1371/journal.pgph.0005262 (PMC12970883; doi:10.1371/journal.pgph.0005262)
Supplement: Attachment — Submitted filename: Response to Reviewers.docx [file pgph.0005262.s005.docx]

**Response to Reviewers**

Dear Editors and Reviewers,

We sincerely thank the reviewers for their thoughtful and constructive comments. We have carefully revised the manuscript in response to each point raised. Below, we provide a detailed, point-by-point response, with reviewer comments reproduced in italics and our responses provided below each point.

**Reviewer 1**

Introduction

“The background on the burden of critical illness is thorough… manuscript would benefit from sentences clarifying why previous efforts… and why now?”

We thank the reviewer for this helpful suggestion. We have now expanded the introduction to explain why previous global critical care priority-setting exercises did not produce an EECC-specific agenda and why the timing of this initiative is appropriate.

“For non-specialist readers, clarify EECC from other advanced critical care definitions.”

We have expanded the introduction to explicitly describe what EECC includes and excludes, emphasising that it focuses on simple, lifesaving care and does not encompass advanced procedures.

Methods

“Describe how thematic groups were assigned… balance of expertise, gender, or regional background.”

We added a clarifying sentence noting that the research team deliberately distributed participants across groups to ensure balanced representation.

“Clarify whether all 46 participants were involved in every round… report completion rates.”

This has been clarified at the beginning of the Participants paragraph, noting minimal attrition and high completion rates.

“Rationale for tool choices (e.g., SmartSurvey).”

We added a sentence describing the rationale (familiarity, usability, and accessibility) and noted that no technical issues occurred.

Results

“Add percentages next to raw numbers in Table 1.”

Table 1 has been revised accordingly.

“Include SD/IQR or comments on question endorsement.”

Our focus was on identifying a top ten set of priorities rather than quantifying the degree of consensus, as noted in the response.

“Explain why no top-ranked questions emerged from the ‘refining’ theme.”

This point is now addressed in the Discussion.

Discussion

“Be more explicit about country representation and absence of patient/community voices.”

We revised the manuscript to quantify the number of countries represented and to explicitly acknowledge the absence of patient or community perspectives.

“Expand discussion of how criteria, facilitation, or digital tools may have shaped dissent or participation.”

A reflective paragraph has been added that addresses potential influences of hierarchy, group dynamics, and digital platforms, along with strategies to mitigate them in future exercises.

“Add concrete suggestions for broadening engagement and integrating equity/feasibility/urgency.”

The final sentence has been rewritten to incorporate these suggestions formally.

Stylistic and Minor Points

“Lines 81–82… specify ‘a consensus-driven exercise…’”

Revised as suggested.

“Reorder current-state questions (Lines 154–159).”

Reordered as recommended.

Ethics and Data

“Add references to other consensus exercises supporting no requirement for formal ethical review.”

Appropriate examples have been added.

“Upload supplementary materials with full list of 28 questions… raw scores.”

Our aim was to produce a ranked list rather than provide detailed quantitative scoring; this is clarified.

**Reviewer 2**

Abstract and Introduction

“What does EECC describe? Is it a course, equipment, protocol?”

We expanded the descriptions in the introduction and main text, clarifying that EECC comprises clinical processes, readiness requirements, tools, and training materials.

“What is the current model of EECC?”

This has been clarified and introduced earlier in the manuscript.

Study Design and Participants

“Total number of conference participants? Was 46 all or selected?”

We added details explaining total attendance, inclusion criteria, and selection of the 46 participants.

“Define relevant clinical backgrounds and research interests.”

We clarified that backgrounds and interests were relevant to emergency and critical care, and have added a supplementary appendix detailing all expert backgrounds (Supplemental Material 1).

“Distribution of expertise across groups—heterogeneous or homogeneous?”

We added a sentence describing the deliberate distribution of expertise to achieve balanced groups.

Prioritisation Process

“Clarify the ‘online document’.”

We clarified that SmartSurvey was used for data collection and that all responses were anonymous.

“Who decided on selecting seven questions per theme?”

A clarifying sentence has been added, noting that research leads proposed seven per group to maintain equal representation across themes.

Ethics

“Was the online document anonymous?”

Yes — this is now stated explicitly.

Discussion

“Consider extension via electronic media; why not a full Delphi?”

We added a statement noting that time constraints prevented a multi-round Delphi, but that future reviews could incorporate extended electronic input.

**Reviewer 3**

“More in-depth review of existing literature to frame relevance.”

Additional references and framing regarding prior critical care priority-setting and the distinctiveness of EECC have been added to the introduction.

“Limited scope of expert panel; provide more details on clinical areas and scope of practice; include supplemental data.”

We agree and have added an explicit reference to the supplementary material, which now includes detailed biographical information for all experts (Supplemental Material 1).

We thank all reviewers again for their insightful comments, which have substantially improved the manuscript. We believe that the revised version is significantly strengthened.

Sincerely,

Ryan Ellis

On behalf of all co-authors
