# Supplementary material for: Top ten research priorities for Essential Emergency and Critical Care: A modified Delphi process
Source: PLOS Glob Public Health. 2026 Mar 9;6(3):e0005262. doi: 10.1371/journal.pgph.0005262 (PMC12970883; doi:10.1371/journal.pgph.0005262)
Supplement: Attachment — Submitted filename: Response to Reviewers_2.docx [file pgph.0005262.s006.docx]

**Response to Reviewers**

Dear Editors and Reviewers,

We sincerely thank the reviewers for their thoughtful and constructive second-round comments. We have carefully revised the manuscript in response to each point raised. Below, we provide a detailed, point-by-point response, with reviewer comments reproduced in italics and our responses provided below each point. We hope this second response now fulfils all criteria for publication.

**Reviewer 1**

***“****The authors must create a supplementary table providing the quantitative scoring data for all 28 questions. Furthermore, the Data Availability Statement on pages 2-3 must be revised to accurately reflect that survey responses (Likert scores) exist, even if only aggregated statistics are being shared. “*

Data has now been extracted from the online survey and collated into a table. This table retains the original question numbers but is ordered by rank, with the top 10 highlighted for clarity. The ranking is based on the mean Likert score. Additional requested data included: SD, median and IQR. This table is included as Supplementary File 2 and is now explicitly mentioned in the main manuscript. The Data Availability Statement has also been updated to reflect the availability of this table in the supplementary table.

*“Geographic and Stakeholder Representation****:*** *The authors must explicitly state the 9-country limitation (primarily high-income settings) in the abstract, as suggested in the first round. In the discussion, the authors should explain why key stakeholders, such as patient and community representatives, were excluded from the initial priority-setting process.”*

The nine-country limitation, which explicitly mentions predominantly high-income countries, is now included in the abstract.

Paragraph added in the discussion section regarding stakeholder involvement. There is now an offered reason for why no patient and community representatives were involved this time, with an honest, reflective statement on the importance of future involvement.

*“Refinement Theme Absence: The discussion on why no top-ranked questions emerged from the 'refining' theme remains superficial. The authors need to critically acknowledge the potential for selection bias, as inviting experts already invested in the existing EECC framework may have influenced the lack of refinement priorities.”*

This paragraph in the discussion section has now been substantially revised, with greater awareness of and critical reflection on potential bias. The closing part of the paragraph maintains that future development may place greater emphasis on this theme, but now also includes the need for participants who are less invested in the original framework.

*“Methods Description: The description of the selection process, where "Each group selected the seven most important research questions," is incomplete. The authors must clarify the mechanism used for this selection-e.g. was it consensus, voting, or individual ranking within the groups?”*

*Added to methods. Revision of Session 4 to address the comment from Reviewer 2 on “why 7 questions?” and stated group selection of questions by consensus.*

*“Critical Reflection on Methods: The discussion on the methods potential impact on results remains minimal. The authors should offer a more critical reflection on: a. Whether dominance patterns were observed during initial in-person group discussions. b. How the compressed two-day format may have limited deep deliberation on complex, cross-cutting issues. c. The potential silo effects created by thematic grouping.”*

Good comments. We accept the limitations of the methods employed. Specific comments in the limitations section of the discussion have now been made regarding these points, including alternative processes proposed as part of the reflective discussion.

*“Equity, Feasibility, and Urgency: The manuscript mentions these important criteria in the conclusion but must explicitly acknowledge as a limitation that these factors were not systematically integrated into the scoring or prioritization framework of this study.”*

It is noted in the discussion section that these criteria were not integrated. The conclusion states that “future priority-setting processes” should include these.

**Reviewer 2**

*“Study Design and Participants
Total number of conference participants? Was 46 all or selected?”
We added details explaining total attendance, inclusion criteria, and selection of the 46
participants.
Comment: Not clear whether total attendees were 46 or more. State this explicitly if this was the case. You mention vast majority completed. What number out of 46 did actually complete. Be explicit (line 105-107)”*

*Now explicit in the text, 46 attended and 41 completed the exercise.*

*“Prioritisation Process*

*“Who decided on selecting seven questions per theme?”
A clarifying sentence has been added, noting that research leads proposed seven per group
to maintain equal representation across themes.
Comment: Noted but not clear why the number 7 was chosen by research leads. Is it based on some prior research? What is the basis for 7?”*

This was a pragmatic decision made by conference leads and agreed upon by participants. Paragraph added to methods as part of Session 4 description to clearly outline this.
