# Supplementary material for: Correction: Top ten research priorities for Essential Emergency and Critical Care: A modified Delphi process
Source: PLOS Glob Public Health. 2026 Sep 29;6(9):e0007403. doi: 10.1371/journal.pgph.0007403 (PMC13623107; doi:10.1371/journal.pgph.0007403)
Supplement: Supplementary file 1 [file pgph.0007403.s001.docx]

**Supplementary File 1. Expert participants**

This supplement lists the experts who participated in the Essential Emergency and Critical Care (EECC) global research priority-setting exercise, along with brief biographical summaries. These details were drawn from the conference participant booklet provided to attendees.

**Organising Committee**

Tim Baker – Associate Professor, Critical Care Physician and Anesthesiologist with positions at Muhimbili University of Health and Allied Sciences in Tanzania and Karolinska Institutet in Sweden; over 20 years’ experience in global critical care research and implementation. Worked with WHO, World Bank, USAID, WFSA and the Centre for Global Development.

Christoffer Hintze – Resident doctor at Karolinska University Hospital with a Bachelor’s in Business and Economics. Extensive experience in strategic healthcare management.

Anna Hvarfner – Medical doctor and PhD student in Global Public Health at Karolinska Institutet; engaged in EECC research with experience from Tanzania and Sweden.

Martin Gerdin Warnberg – Associate Professor of Clinical Epidemiology in the Department of Global Public Health at Karolinska Institutet and a Resident in Anaesthesia and Intensive Care at Karolinska University Hospital. Lead the ‘Improving Trauma Systems’ team within the Health Systems and Policy research group.

**Expert Participants**

Kun Arifi Abbas – Anaesthesiologist and Intensivist at Dr Soetomo General Hospital Surabaya, Indonesia. Also a lecturer at the Anaesthesiology and Reanimation Department at the Faculty of Medicine Universitas Airlangga, Indonesia.

Opeolu Adeoye – Professor and Chair of the Department of Emergency Medicine at the Washington University School of Medicine in St. Louis, Missouri. Emergency Physician-in-Chief at Barnes-Jewish Hospital. Serves on the Editorial Board of Stroke Journal, and is an American Heart Association Stroke Section Co-Editor.

Tobias Alfven – Clinical Professor in Global Child Health at Karolinska Institutet and senior consultant in paediatrics at Sachs’ Children and Youth Hospital. Co-leads the Centre of Excellence for Sustainable Health for colleagues from Uganda. Also President of the Swedish Society of Medicine from 2020-2024.

Helle Alvesson – Trained as a medical anthropologist, now at the Department of Global Public Health, Karolinska Institutet, Sweden. Expert in qualitative and participatory methods with a focus on scaling health interventions in low-resource settings.

Wurry Ayuningtyas – Paediatrician from Surabaya, Indonesia. Currently undergoing a trainee year to specialise in neonatology at Soetomo General Hospital, Indonesia.

Ulrika Baker – Medical doctor and public health specialist with over 20 years of experience in clinical practice, research and public health. Leads UNICEF’s work on strengthening the national Primary Health Care system to deliver quality services to mothers, newborns and children.

Godfrey Barabona – Experienced researcher and clinician based in Tanzania. Active member of EECC research team and Tanzania national lead for the African Partnership for Perioperative and Critical Care Research.

Andreas Barratt-Due – Senior consultant in anesthesiology and critical care, serving as medical head of the ICU at Rikshospitalet, Oslo University Hospital. Six years’ experience with the Department of Global Health at Oslo University Hospital.

Temesgen Beyene – Associate Professor and Consultant in Emergency Medicine and Critical Care at Addis Ababa University, Ethiopia. International Fellow for the Institute for Healthcare Improvement focused on Quality. Past Chair of the Department of Emergency Medicine at the College of Health Sciences, Tikur Anbessa Specialised Hospital in Ethiopia.

Jonas Blixt – Neuro ICU clinician and previous Director of Intensive Care at Karolinska University Hospital, Sweden. Research focusing on improving care is post-traumatic brain oedema with experience across global settings.

Dan Brun – Emergency Physician and public health specialist currently acting Chief Executive Officer and Medical Director at Holbaek Hospital, Denmark. Previously senior health policy advisor at the Ministry of Health in Tanzania. Involved in national response to COVID-19 in both Tanzania and Denmark.

Mariam Claeson – Honorary medical doctor and senior advisor at Karolinska Institutet. Worked for many years across reproductive, maternal, newborn, child, and adolescent health, as former director of the Global Financing Facility for Women, Children and Adolescents at the World Bank. Also at the Maternal Newborn and Child health team at the Gates Foundation. Co-editor of the 2nd Disease Control Priorities and commissioner of the Lancet Commission on Adolescent Health.

Louise Elander – Senior consultant in Anaesthesia and Intensive Care at Nyköping Hospital, Sweden.

Birger Forsberg – Associate Professor in International Health at Karolinska Institutet. Medical doctor with a speciality in Social Medicine. Main fields of work in health planning, evaluation and health economics in Sweden and internationally. Work experience as a staff member and consultant with WHO, the World Bank and UNICEF. Research in communicable diseases, child health and health system issues.

Claudia Hanson – Specialist in Obstetrics and Gynaecology and Professor of Implementation Science and Perinatal Epidemiology at Karolinska Institutet, Sweden and the London School of Hygiene and Tropical Medicine, UK. Leads a research group on implementation science and quality at Karolinska Institutet.

Mats Holmberg – Associate Professor at Linnaeus University and an R&D director at the ambulance department at Region Sörmland. Research covers prehospital emergency care from interpersonal and ethical perspectives.

Kjell Arne Johansson – Professor of medical ethics at the Bergen Centre for Ethics and Priority Setting at the University of Bergen. Focuses on developing frameworks for fair prioritisation in healthcare. and health economics, University of Bergen. Research centres around evidence-based decision-making and ethical guidance of resource allocation.

Aneth Kaliza – Medical doctor and researcher at Muhimbili University of Health and Allied Health Sciences. Part of the research team in Tanzania with interest in policy reform and accessible critical care.

Karima Khalid – Lecturer and consultant in Intensive Care at Muhimbili University of Health and Allied Sciences in Tanzania. Member of Tanzania Ministry’s NSOAP technical working group.

Carina King – Associate Professor of Global Health at the Karolinska Department of Global Public Health. Co-lead of the Lancet Global Health Commission on Medical Oxygen Security, and co-chair of the Every Breath Counts research group.

Teresa Bleakly Kortz – Associate Professor of Clinical Paediatrics in Critical Care Medicine, Director of the Institute for Global Health Sciences Affiliate Program, and Director of the Paediatric Global Health Pathway at the University of California, San Francisco. NIH-funded researcher, WHO sepsis consultant and lead for hr UCSF-WHO Collaborating Centre.

Margaret E. Kruk – Professor of Health Systems at the Harvard T.H. Chan School of Public Health and incoming Distinguished Professor of Health Systems and Medicine in the School of Medicine at Washington University in St. Louis. Uses implementation science and econometric methods to design and evaluate large-scale health system reforms.

Nick Leech – Oversees media for GoDaddy. Previously led marketing for Host Europe Group. Leads communications for the EECC global network.

Miklos Lipsey – Professor and Consultant in Anaesthesiology and Intensive Care at Uppsala University, Sweden.

Halinder Mangat – Dual certified in neurology and internal medicine and Associate Professor in the Department of Neurology and a neurocritical specialist with The University of Kansas Health System. Extensive experience in capacity building in Tanzania with continued interest in global neurotrauma.

Alhassan Datti Mohammed – Consultant in Anaesthesiology and Intensive Care at the Aminu Kano Teaching Hospital and Associate Professor of Anesthesiology at Bayero University, Kano, Nigeria.

Celia Blaas Kia Ora – Resident Anaesthesiologist from New Zealand. Completed a Master’s in Global Health from Karolinska and continued working in the Department of Global Health.

Kumanan Rasanathan – Executive Director of the Alliance for Health Policy and Systems Research at WHO in Geneva, Switzerland. Public Health physician with background in health policy and systems research. Served as an elected board member of Health Systems Global from 2016-2020.

Ava Rimdahl – Pursuing a master’s degree in International Humanitarian Action at Uppsala University. Academic background in communication with a focus on the intersection of advocacy, global health, and humanitarian action.

Nobhojit Roy – Trained as a General and Trauma surgeon from Mumbai. Professor and Chair of the Centre for Leadership at the University of Global Health Equity, Rwanda. Lead of the Program in Global Surgery and Trauma at The George Institute of Global Health, India. Also Technical Officer for Surgery and Anaesthesia at the WHO Headquarters, Geneva and National Advisor to the Ministry of Health and Family Welfare, India.

Petronella Bjurling-Sjöberg –Background as a specialised ICU nurse. PhD in Medical Science, currently Patient Safety Coordinator and Associate Researcher at Centre of Clinical Research, Sörmland.

Ryan Ellis – Anesthesiology resident in the UK and member of the African Partnership for Perioperative and Critical Care Research (APPRISE).

Jamie Rylance – Physician in pulmonary and internal medicine. Works within the WHO Emergencies Programme, providing support for clinical management and guidelines in health emergencies. Previous research experience across Malawi and Tanzania.

Carl Otto Schell – Senior Consultant Physician in Internal Medicine and Cardiology at Nyköping Hospital in Sweden. Researcher at Global Public Health at Karolinska Institutet and the Centre for Clinical Research Sörmland, Uppsala University, Sweden. Global research encompasses the epidemiology of critical illness and the potential to strengthen resilient health systems. Work involves collaborations with UNICEF, WHO and the World Bank.

Simon-Fredrick Schell – Medical student studying at Uppsala University. Active with the International Federation of Medical Students.

Save Shroder – Anaesthesiologist at the Juliane Marie Centre at the Rigshospital in Denmark. Part of the Nordic Network for Global Surgery.

Septo Sulistio – Emergency physician and Anaesthesiologist at Cipto Mangunkusuma National General Hospital (RSCM) in Indonesia. Acts as head of the pre-hospital emergency services at RSCM.

Emily Tegnell – Specialist in Anaesthesiology and Intensive Care Medicine at the Sahlgrenska Hospital in Gothenburg, Sweden. Global health experience including EECC implementation in Zambia.

Andreas Wellhagen – Specialist in Anesthesiology and Intensive Care at Nyköpings Lasarett Hospital, Sweden. Member of EECC Sweden Hub with research projects within EECC themes.

Alexandra Wharton-Smith – Global health researcher and technical adviser with a BA Hons in International Relations; PhD candidate at LSHTM. Co-led the global consensus Delphi on EECC treatments and actions.

Nora Zergi – Specialist in Anesthesiology and Intensive Care at Haukeland University Hospital, Norway.
